# Are there racial/ethnic differences in antibiotic treatment of community acquired pneumonia in the inpatient setting?

**DOI:** 10.1371/journal.pone.0345788

**Published:** 2026-03-25

**Authors:** Shana A. B. Burrowes, Mari-Lynn Drainoni, Tamar F. Barlam

**Affiliations:** 1 Section of Infectious Diseases, Department of Medicine, Boston Medical Center & Boston University Chobanian and Avedisian School of Medicine, Boston, Massachusetts, United States of America; 2 Evans Center for Implementation and Improvement Sciences, Department of Medicine, Boston University Chobanian and Avedisian School of Medicine, Boston, Massachusetts, United States of America; 3 Department of Health Law Policy and Management, Boston University School of Public Health, Boston, Massachusetts, United States of America; Emory University, UNITED STATES OF AMERICA

## Abstract

**Background:**

Community Acquired Pneumonia (CAP) is the most common reason for antibiotic treatment in hospitalized adults. Some prior studies have found treatment differences by race/ethnicity, but research on the topic is limited, results are mixed, and it is unclear if clinical outcomes are affected.

**Methods:**

We conducted a retrospective analysis of hospitalized patients >=18 years of age with a diagnosis of CAP from 2018–2021 across 457 US hospitals in the Vizient Inc. Clinical Data Base. We examined guideline concordant antibiotic treatment differences for inpatient CAP by patient race/ethnicity and hospital level factors and secondarily assessed whether treatment differences affect patient clinical outcomes.

**Results:**

There were 1,277,770 admissions. Over half of all patients received concordant antibiotic therapy. Non-Hispanic Black patients had an increased odds of receiving guideline concordant antibiotic care (OR 1.22 95% CI 1.21–1.23) compared to non-Hispanic White patients. As the number of hospital beds increased, the odds of receiving concordant therapy decreased, with the greatest reduced odds between hospitals with >500 beds vs < 75 beds (OR 0.68, 95% CI 0.66–0.70). As case mix index increased concordant care decreased with the lowest odds observed at hospitals with a case mix index >2 (OR 0.71 95% CI 0.7–0.72). Patients at hospitals in the South (OR 0.8 95% CI 0.79–0.81) and Northeast (OR 0.71, 95% CI 0.7–0.72) were less likely than those in the West to receive concordant care. There was no significant association between the interaction of race/ethnicity and receipt of guideline concordant therapy and clinical outcomes.

**Conclusion:**

Non-Hispanic Black patients were more likely to received guideline-concordant care for CAP however significant differences in concordant therapy were seen at the hospital level. Understanding the interplay of race/ethnicity and concordant CAP therapy at the individual and population level is important for future research in the examination of disparities in care.

## Background

Community acquired pneumonia (CAP) is one of the most common reasons why inpatients receive antimicrobial treatment [1]. Studies have shown that antibiotic treatment for CAP is often inappropriate [1–4] (e.g., non-guideline concordant antibiotic or excessive duration of antibiotics) which can lead to negative patient outcomes, such as treatment failure, and adverse events such as allergic reactions, antibiotic resistance and *Clostridioides difficile* infections [2,5,6]. In the outpatient setting, studies show that patients from racial/ethnic minority groups receive different antibiotic treatment compared to their White counterparts [7,8]. This includes being prescribed fewer antibiotics, receiving different antibiotics for the same disease and experiencing longer time to receipt of treatment [7–12]. However, research on the influence of racial/ethnic differences on inpatient antibiotic prescribing for CAP is more limited. One study found that White inpatients with pneumonia are treated more quickly with antibiotics than Hispanic or African American patients [12]. Another study by Frei et al. reported reduced likelihood of receipt of guideline concordant treatment in Black patients compared to White patients [13]. Results from studies that have examined racial/ethnic differences in clinical outcomes (e.g., mortality, length of stay etc.) in CAP patients have been mixed [12,14,15] and the role that antibiotic treatment differences may play is unknown. In the current study, we take a comprehensive approach to assessing differences in the antibiotic treatment of CAP. We examined treatment differences by race/ethnicity, as well as hospital level factors and evaluated associations between any treatment differences and four patient clinical outcomes: length of stay >7 days, 30-day readmission, adverse events, and clinical complications and mortality.

## Methods

### Data source

We obtained data from Vizient Inc., the third largest healthcare performance company in the US [16]. The Vizient Clinical Data Base houses data on clinical benchmarks and resource utilization for over 1000 facilities including academic medical centers, community hospitals and essential and non-essential hospitals. The Vizient Clinical Data Base contains facility demographic data such as case mix index and hospital ownership status and patient-level data uploaded voluntarily each quarter by participating Vizient institutions and allows for comparison of clinical outcomes across institutions. Only continuously reporting hospitals that submitted data during the study period were included. Access to the Vizient Clinical Data Base is granted through Boston Medical Center’s license agreement. Data was accessed from Vizient on 01/05/2023. Data from the Vizient Clinical Data Base were used by permission of Vizient, Inc. All rights reserved.

### Study variables

We analyzed data on all hospitalized adult patients >=18 years of age with a diagnosis of CAP (ICD10 codes: J13-J18), from 01/01/2018–12/31/2021. We did not include COVID-19 pneumonias. For the cohort, we collected the following demographic information from the database: age, race, ethnicity, sex, insurance type. Race/ethnicity was captured in the following categories as reported by Vizient: non-Hispanic Black, non-Hispanic White, Hispanic, Asian and Other.

Clinical data extracted included specific antibiotics received, clinical comorbidities (e.g., COVID-19, comorbidities contained in the Elixhauser comorbidity index) [17], adverse events/infections (e.g., *C. difficile, Methicillin-resistant Staphylococcus aureus (MRSA)*, length of stay, ICU stay (yes/no), mortality (defined as died-in-hospital). In addition to data on the patient cohort, we collected information on the following hospital factors: case mix index (CMI), number of hospital beds, and geographic location. CMI was provided directly from Vizient, includes all relevant severity diagnosis groups and is updated quarterly. CMI was categorized as follows: < 1.6, 1.6–1.8, > 1.8–2 and >2. Higher CMI values indicate hospitals with underlying patient populations of higher diversity, complexity and severity. The number of hospital beds was categorized as follows: < 75, 75–199, 200–499 and>=500 beds.

### Outcome measures

The primary outcome was receipt of antibiotic therapy concordant with 2019 American Thoracic Society/Infectious Diseases Society of America Clinical Practice Guidelines for CAP [18]. Receipt of concordant therapy was defined as combination therapy with a β-lactam (ampicillin + sulbactam, cefotaxime, ceftriaxone or ceftaroline) and a macrolide (azithromycin or clarithromycin) or a β-lactam and doxycycline (first line) or monotherapy with a respiratory fluoroquinolone (levofloxacin or moxifloxacin) (second line). In this study we did not include time to antibiotics or duration of therapy as part of our definition for guideline concordance.

We examined the following four secondary clinical outcomes: (1) length of stay >7 days, (2) 30-day hospital readmission, (3) adverse events or complications, (4) mortality. Adverse events included anemia, *C. difficile*, hypotension, MRSA, MRSA pneumonia, *non-C. difficile* diarrhea, resistance to other antibiotics, rash, sepsis due to MRSA, thrombocytopenia. Vizient complications are defined by Vizient to give a comprehensive array of complications that could occur in the healthcare setting. Vizient includes 13 complications among the adult medical-surgical patient population and of those we included the following which were relevant to our study: readmissions for infection due to previous care, aspiration pneumonia, post-operative infection, hospital acquired *C. difficile enteritis*. In the final analytical dataset, we created a composite outcome defined as experiencing either an adverse event and/ or complication.

### Statistical analysis

Univariate and bivariate analyses were conducted prior to building the regression model and examined the distribution of patient demographic and clinical characteristics as well as hospital factors by race/ethnicity utilizing parametric (Chi Square and t-tests) and non-parametric tests (Wilcoxon and Kruskal Wallis). Continuous variables are reported with medians and interquartile ranges and categorical variables are reported with frequencies and percentages. Due to our large sample size, potential confounders were initially assessed with an OR>= 1.2 or OR <= 0.8 and p ≤ 0.05 based on their association with race/ethnicity and receipt of guideline concordant antibiotic therapy; those that met these criteria were eligible for inclusion in the final model as explained below. Additionally, confounders were assessed based on clinical relevance and biological plausibility such as age, sex and clinical comorbidities (e.g., diabetes).

Exploratory analyses examined first and second line treatment separately. However, we found no differences between first and second line treatments and the final models defined concordant care as receiving either first or second line therapy. Final models examined the relationship between race/ethnicity and guideline-concordant antibiotic treatment. Confounders were added to the models one at a time (based on prior criteria where those with the strongest association based on OR were added to the model first) and assessed for their impact on the primary association between race/ethnicity and guideline-concordant antibiotic treatment followed by variables that were biologically relevant. To assess for multicollinearity between variables, we first examined correlations between predictors prior to building the regression model, then as each variable was added to the regression model, we examined (1) how much of the variance it explained and (2) its impact on the other variables in the model with which it was known to be correlated. We compared models with the presence of both variables to the model with each variable separately. For example, COVID-19 and year were highly correlated however, year accounted for additional variance not explained by a COVID-19 diagnosis and improved the overall fit of the model and thus we kept year in the model. Due to differences in treatment options associated with MRSA, we also examined the role of MRSA in the model. However, this was not significant and only 0.13% (n = 1620) patients had a MRSA diagnosis. Additionally ICU admissions was assessed as a potential confounder in the model as admission to the ICU indicates severity of illness and can impact treatment choice. We also examined interactions between race/ethnicity and each of the following factors in separate models: renal failure, cerebrovascular disease, case mix index, geographic region. However, these were not significant and were not included in the final model. Given existing literature around the use of broad-spectrum antibiotics in CAP treatment, additional analyses examined the relationship between race/ethnicity and receipt of broad-spectrum antibiotics (vancomycin, linezolid, ertapenem, imipenem/cilastatin, piperacillin/tazobactam, meropenem, tigecycline, cefepime), but there was no significant association. The final model included all variables listed in Table 3.

Secondary outcome analyses examined the interaction between race/ethnicity and concordant antibiotic treatment with length of stay >7 days (yes/no), 30-day hospital readmission (yes/no), adverse event or complication (yes/no) and mortality (yes/no) in separate models. For all analyses, we used hierarchical multivariable regression models operationalized through generalized estimation equations to account for clustering within patients and among patients hospitalized at the same facility. All analyses used SAS (v.9.4, SAS Institute Inc. Cary, NC). The study adheres to STROBE reporting guidelines for observational studies. The institutional review board at Boston University Medical Campus and Boston Medical Center approved these analyses. The study does not include factors necessitating patient consent as all data was de-identified and fully anonymized before it was accessed.

## Results

### Sample description

There were 1,277,770 admissions of patients with CAP across 457 hospitals over the four-year period from 2018–2021. Table 1 shows a description of the patient sample by race/ethnicity. The sample was predominantly White (n = 882,691, 69.1%) and just over half were male (n = 663,175, 51.9%). Overall patients were primarily insured by Medicare (n = 821,680, 64.3%); this ranged from 46.7% in Hispanic patients to 69.7% in non-Hispanic White patients. Non-Hispanic White and Asian patients were older (70 and 71 years respectively) than other racial and ethnic groups who were between 61–63 years old. Non-Hispanic Black patients had higher proportions of hypertension (n = 161,195, 70.36%) and renal failure (n = 77,550, 33.85%) and non-Hispanic White patients had a higher proportion of chronic pulmonary disease (n = 407,207, 46.13%) compared to other racial/ethnic groups. Almost half of patients were seen at large hospitals >500 beds (n = 613,071, 47.98%), with a CMI > 2 (n = 688,194, 53.86%). Most patients in our sample were seen at hospitals located in the South (n = 431,342, 33.76%) or Midwest (n = 427,659, 33.47%). Fifty percent (n = 116,178) of non-Hispanic Black patients were seen at hospitals in the South and 38.43% (n = 339,188) of non-Hispanic White patients were seen at hospitals in the Midwest (Table 2). Hospital level data by region is presented in S1 Table. Briefly, 37% of hospitals were located in the Midwest and 29% were located in the South; 38.6% and 32% of hospitals in the Northeast and the South respectively had >=500 beds. In all regions except the Midwest, hospitals with a CMI > 2 accounted for over 35% of hospitals in the region.

**Table 1:** Demographic and clinical characteristics of admissions with a primary diagnosis of community acquired pneumonia 2018-2021.

Variable	All n = 1277770	Hispanic/ 94582 (7.4%)	Non-Hispanic Black/ 229091 (17.93%)	Non-Hispanic White/ 882691 (69.08)	Asian/ 29900 (2.34%)	Other/ 41506 (3.25%)
**Sex/n (%)**						
Female	614559 (48.1)	42735 (45.18)	115833 (50.56)	423631 (47.99)	13320 (44.55)	19040 (45.88)
Male	663175 (51.9)	51845 (54.82)	113252 (49.44)	459037 (52.01)	16580 (55.45)	22461 (54.12)
**Age/ median±IQR**	68 ± 23	62 ± 27	61 ± 22	70 ± 21	71 ± 23	63 ± 25
**Insurance Type/n (%)**						
Commercial	202124 (15.82)	14744 (15.59)	33391 (14.58)	140639 (15.93)	6119 (20.46)	7231 (17.42)
Medicaid	181857 (14.23)	25074 (26.51)	54581 (23.83)	86933 (9.85)	4974 (16.64)	10295 (24.8)
Medicare	821680 (64.31)	44121 (46.65)	124157 (54.2)	615033 (69.68)	17610 (58.9)	20759 (50.01)
Other	72109 (5.64)	10643 (11.25)	12962 (7.4)	40086 (4.54)	1197 (4)	3221 (7.76)
**COVID-19/ n (%)**	52875 (4.14)	7517 (7.95)	10428 (4.55)	30679 (3.48)	1582 (5.29)	2669 (6.43)
**Elixhauser Comorbidities/ n (%)**						
Cerebrovascular disease	70170 (5.49)	4732 (5)	16633 (7.26)	44411 (5.03)	1982 (6.63)	2412 (5.81)
Chronic Pulmonary Disease	547560 (42.85)	27971 (29.57)	90034 (39.3)	407207 (46.13)	8575 (28.68)	13773 (33.18)
Diabetes with/without Complications	454393 (35.56	43020 (45.48)	92770 (40.49)	288708 (32.71)	12681 (42.41)	17214 (41.47)
Diabetes with chronic complications	324570 (25.4)	30647 (32.4)	69018 (30.13)	204032 (23.11)	8667 (28.9)	12206 (29.41)
Diabetes without chronic complications	129823 (10.16)	12373 (13.08)	23752 (10.37)	84676 (9.59)	4014 (13.42)	5008 (12.07)
Heart Failure	444663 (34.8)	27643 (29.23)	84402 (36.84)	310860 (35.22)	8692 (29.07)	13066 (31.48)
Hypertension	845399 (66.16)	57906 (61.22)	161195 (70.36)	581105 (65.83)	19744 (66.03)	25449 (61.31)
Hypertension complicated	474322 (37.12)	32173 (34.02)	96678 (42.2)	320667 (36.33)	10649 (35.62)	14155 (34.1)
Hypertension uncomplicated	371077 (29.04)	25733 (27.21)	64517 (28.16)	260438 (29.51)	9095 (30.42)	11294 (27.21)
**Variable**	**All n = 1277770**	**Hispanic/ 94582 (7.4%)**	**Non-Hispanic Black/ 229091 (17.93%)**	**Non-Hispanic White/ 882691 (69.08)**	**Asian/ 29900 (2.34%)**	**Other/ 41506 (3.25%)**
Obesity	257417 (20.15)	20207 (21.36)	52559 (22.94)	174847 (19.81)	1777 (5.94)	8027 (19.34)
Pulmonary circulation disease	126377 (9.89)	7685 (8.13)	27722 (12.1)	84567 (9.58)	2594 (8.68)	3809 (9.18)
Renal Failure	357041 (27.94)	26905 (28.45)	77550 (33.85)	232274 (26.31)	8957 (29.96)	11355 (27.36)
Moderate renal failure	226985 (17.76)	12575 (13.3)	39387 (17.19)	164253 (18.61)	4739 (15.85)	6031 (14.53)
Severe renal Failure	130056 (10.18)	14330 (15.15)	38163 (16.66)	68021 (7.71)	4218 (14.11)	5324 (12.83)
Liver disease	125286 (9.81)	12951 (13.69)	23504 (10.26)	80257 (9.09)	3281 (10.97)	5293 (12.75)
Mild liver disease	94182 (7.37)	8948 (9.46)	19879 (8.68)	59215 (6.71)	2528 (8.45)	3612 (8.7)
Moderate/severe liver disease	31104 (2.43)	4003 (4.23)	3625 (1.58)	21042 (2.38)	753 (2.52)	1681 (4.05)
**ICU during index admission/n (%)**	487179 (38.13)	33428 (35.34)	90017 (39.29)	336406 (38.11)	11416 (38.18)	15912 (38.34)
**Concordant Antibiotic Therapy/n (%)**	719329 (56.3)	55234 (58.4)	132172 (57.69)	492697 (55.82)	16359 (54.71)	22867 (55.09)
**Complication or Adverse Event** ^**a**^ **/n (%)**	100410 (7.86)	7198 (7.61)	18748 (8.18)	68144 (7.72)	2544 (8.51)	3776 (9.1)
**Readmission within 30 days/ n (%)**	191018 (14.95)	14490 (15.32)	36755 (16.04)	129590 (14.68)	4383 (14.66)	5800 (13.97)
**Died in hospital/n (%)**	110393 (8.64)	8613 (9.11)	18113 (7.91)	75755 (8.58)	3342 (11.18)	4570 (11.01)
**Length of stay days/n (%)**						
<=7	866805 (67.84)	61367 (64.88)	148508 (64.83)	610883 (69021)	19706 (65.91)	26341 (63.46)
>7	410964 (32.16)	33215 (35.12)	80582 (35.17)	271808 (30.79)	10194 (34.09)	15165 (36.54)

a Adverse events include anemia*, Clostridioides difficile*, hypotension, Methicillin-resistant *Staphylococcus aureus*, Methicillin-resistant *Staphylococcus aureus* pneumonia, non-*Clostridioides difficile* diarrhea, resistance to other antibiotics, rash, sepsis due to Methicillin-resistant *Staphylococcus aureus*, thrombocytopenia.

a Vizient complications include readmissions for infection due to previous care, aspiration pneumonia, post-operative infection, hospital acquired *Clostridioides difficile enteritis*

**Table 2:** Description of hospital characteristics of admissions with a primary diagnosis of community acquired pneumonia 2018-2021.

Hospital Characteristics	All n = 1277770	Hispanic/ 94582 (7.4%)	Non-Hispanic Black/ 229091 (17.93%)	Non-Hispanic White/ 882691 (69.08)	Asian/ 29900 (2.34%)	Other/ 41506 (3.25%)
**Geographic Region/n (%)**						
Midwest	427659 (33.47)	10549 (11.15)	63489 (27.71)	339188 (38.43)	5000 (16.72)	9433 (22.73)
Northeast	241213 (18.88)	19642 (20.77)	32609 (14.23)	169253 (19.17)	7748 (25.91)	11961 (28.82)
South	431342 (33.76)	32481 (34.34)	116178 (50.71)	266623 (30.21)	6692 (22.38)	9368 (22.57)
**Variable**	**All n = 1277770**	**Hispanic/ 94582 (7.4%)**	**Non-Hispanic Black/ 229091 (17.93%)**	**Non-Hispanic White/ 882691 (69.08)**	**Asian/ 29900 (2.34%)**	**Other/ 41506 (3.25%)**
West	177556 (13.9)	31910 (33.74)	16815 (7.34)	107627 (12.19)	10460 (34.98)	10744 (25.89)
**Number of hospital beds/ n (%)**						
<75	57928 (4.53)	4499 (4.76)	4066 (1.77)	47783 (5.41)	504 (1.69)	1076 (2.59)
75-199	197049 (15.42)	11911 (12.59)	23577 (10.29)	152734 (17.3)	4014 (13.42)	4813 (11.6)
200-499	409722 (32.07)	30153 (31.88)	67148 (29.31)	290427 (32.9)	10450 (34.95)	11544 (27.81)
>=500	613071 (47.98)	48019 (50.77)	134300 (58.62)	391747 (44.38)	14932 (49.94)	24073 (58)
**Case Mix Index/ n (%)**						
<1.6	101174 (7.92)	2925 (3.09)	7560 (3.3)	88144 (9.99)	930 (3.11)	1615 (3.89)
1.6-1.8	250495 (19.6)	15945 (16.86)	35820 (15.64)	187843 (21.28)	5514 (18.44)	5373 (12.95)
1.81-2	237907 (18.62)	17584 (18.59)	42820 (18.69)	166203 (18.83)	4645 (15.54)	6655 (16.03)
>2	688194 (53.86)	58128 (61.46)	142891 (62.37)	440501 (49.9)	18811 (62.91)	27863 (67.13)

Over half (n = 719,329) of patients across all racial and ethnic groups received concordant antibiotic therapy (range 54–58%). Over one third (30.7–36.5%) of patients across all racial and ethnic groups had a length of stay >7 days. Readmissions within 30 days ranged from 14–16% (n = 191,018) across all groups. Complications or adverse events ranged from 7.6–9% (n = 100,410) across all groups.

### Primary and secondary outcomes

Table 3 shows that non-Hispanic Black patients had increased odds of receiving guideline concordant antibiotic care (OR 1.22 95% CI 1.21–1.23) compared to non-Hispanic White patients. There was no significant difference in receipt of concordant care between Hispanic patients and non-Hispanic White patients. Patients with renal failure (OR 0.76, 95% CI 0.76–0.77) or cerebrovascular disease (OR 0.71, 95% CI 0.70−72) had reduced odds of receiving guideline concordant care regardless of race/ethnicity. As the number of hospital beds increased, the odds of receiving concordant therapy decreased, with the greatest reduced odds between >500 beds vs < 75 beds (OR 0.68 95% CI 0.66–0.69). Similarly, as CMI increased the odds of concordant care decreased with the lowest odds observed at hospitals with a CMI > 2 (OR 0.71 95% CI 0.69–0.72). Patients hospitalized in the South (OR 0.8 95% CI 0.79–0.81) and Northeast (OR 0.71, 95% CI 0.7–0.72) were less likely than those in the West to receive guideline concordant care. Receipt of guideline concordant care decreased over time with the greatest reduced odds in 2021 (OR 0.73, 95% CI 0.73–0.74). See S2 table for regression coefficients and standard errors.

**Table 3:** Association between race/ethnicity and receipt of antibiotic guideline concordant care for community acquired pneumonia.

Variable	
**Race/Ethnicity**	Adjusted Odds Ratio (95% Confidence Interval)^a,b^
Non-Hispanic White	**Reference**
Non-Hispanic Black	1.22 (1.21-1.23)
Asian	1.00 (0.98–1.03) ^c^
Hispanic	1.15 (1.14-1.17)
Other	1.05 (1.03-1.07)
**Cerebrovascular disease**	0.71 (0.70-0.72)
**Renal failure**	0.76 (0.76-0.77)
**Number of hospital beds**	
<75	**Reference**
75-199	0.94 (0.92-0.96)
200-499	0.81 (0.80-0.83)
>=500	0.68 (0.66-0.69)
**Case Mix index**	
<1.6	**Reference**
1.6-1.8	0.84 (0.83-0.85)
>1.8-2	0.93 (0.92-0.95)
>2	0.71 (0.69-0.72)
**Hospital Region**	
West	**Reference**
South	0.8 (0.79-0.81)
Midwest	0.83 (0.81-0.83)
Northeast	0.71 (0.70-0.72)
**Year**	
2018	**Reference**
2019	0.87 (0.86-0.88)
2020	0.80 (0.79-0.81)
2021	0.73 (0.73-0.74)

a Unless indicated all variables had p < .0001

b Statistical significance was assigned at OR>= 1.2 or OR <= 0.8 and p ≤ 0.05

c Indicates p value > 0.05

Secondary analyses found there was no significant association between the interaction of race/ethnicity and receipt of guideline concordant therapy and any of the secondary clinical outcomes.

## Discussion

In this study, we found that Black patients had higher odds of receiving guideline concordant antibiotic therapy compared to White patients, but there were no other differences in guideline concordant care by race/ethnicity. Patient clinical comorbidities and hospital level factors, such as case mix index and number of hospital beds, played a role in receipt of guideline concordant care. We did not find that the interaction between guideline concordant care and race/ethnicity (i.e., treatment differences by race/ethnicity) was significantly associated with any clinical outcomes. This suggests that although we found a statistically significant guideline concordant difference between Black and White patients, it may not be a clinically significant or it may affect other clinical outcomes that we did not address in this study.

Our finding that Black patients had increased odds of receiving guideline concordant care compared to White patients contrasts with recent literature. Frei et al. report found that non-Hispanic White patients had a 36% higher likelihood of receiving guideline concordant care compared to Black patients and that regardless of race, patients of non-Hispanic ethnicity were less likely to receive guideline concordant care compared to Hispanic patients [13]. While that study adjusted for sociodemographic factors such as smoking and alcohol consumption, it did not account for underlying chronic conditions, which may explain some differences in our findings [13]. For example, we found that patients with comorbidities had reduced odds of receiving guidelines concordant therapy regardless of race/ethnicity. This is likely due to the difficulty associated with treating medically complex patients. Although ATS/IDSA guidelines do not explicitly address treatment of CAP in hospitalized adults with comorbidities, some research shows the presence of these may impact treatment decisions. A study conducted across 15 Veterans Affairs (VA) hospitals that examined factors influencing antibiotic prescribing decisions for four common infections including pneumonia found that patient factors were important [19]. Specifically when providers were presented with clinical scenarios some were unable to provide a prescribing decision without more information on the clinical picture, stating that renal function, patient age and underlying comorbidities played an important role [19]. Similar work at three urban medical centers found that when providers were presented with different clinical vignettes physicians were more likely to prescribe non-concordant antibiotics for clinically complex patients (e.g., older age, high medical comorbidity etc.) [20]. In the context of our findings this suggests that when patients present with underlying comorbidities, providers may find it necessary to adjust treatment in a way that they think best serves their patients.

Across the literature other studies have shown that while Black patients may have a longer time to treatment there are no differences in receipt of guideline concordant treatment [15,21]. These studies, however, had restricted inclusion criteria (e.g., older patients only) and were limited to either the VA or a single site [15,21]. A recent study examining the receipt of anti-*Pseudomonas* and anti-MRSA agents in pneumonia patients, adjusting for age, severity and indication, found that use of anti-MRSA agents was similar across all racial and ethnic groups [22]. However, younger non-Hispanic Black patients were less likely to receive anti-*Pseudomonas* agents and among any patients receiving anti-P*seudomonas* agents, non-Hispanic Black patients received them for a lower proportion of their visit [22]. The persistence of mixed findings within and across studies suggests a need for research that assesses comprehensive definitions of guideline treatment concordance utilizing several metrics such as antibiotic choice, time to treatment and duration of therapy. It also suggests that observed differences have underlying nuances that require further examination and research should pinpoint findings that are clinically significant to patient outcomes.

There were significant differences by hospital level factors. We found that larger hospitals, those located in the South and Northeast and hospitals with a case mix index >2 were less likely to provide guideline concordant care. The variation in treatment across hospitals could be due to physician practices, specific prescribing culture within a hospital and/or the demographics of the population served by the hospital. For example, Mayr et al. found that after adjustment for CMI there was no difference in guideline concordant therapy between Black and White patients but hospitals that served a greater proportion of Black patients were less likely to provide timely antibiotics [23]. A recent paper by Gathers et al. found that racial and ethnic disparities in hospital mortality of pediatric CAP was driven by geography and age [24]. Among patients <1 year, Black patients in the South (OR 2.35) and West (OR 2.47) and Hispanic/Latino patients in the Northeast (OR 2.36) experienced the highest mortality compared to White patients in the Northeast [24]. This suggests that at the population level race/ethnicity may play a role in antibiotic treatment, even if it is not reflected at the individual level. Although we could not examine race/ethnicity at the hospital level in our analysis, future studies should utilize data such as the social vulnerability index to better capture the underlying population served by the hospital.

At the hospital level it is also important to recognize that centers with large number of beds and high CMI are more likely to deal with complex patients and providers may have made treatment decisions on the individual level [20,19] that was more clinically appropriate and due to the overall acuity of their patient population this was reflected at the hospital level. Additionally research has shown that antibiotic use in the outpatient setting in the South is consistently higher compared to other regions in the US [25–27]. Overall prescribing practices in a region can affect resistance pattern [28]. This may impact local antibiograms at hospitals within the region which may affect provider prescribing decisions in the inpatient setting [29]. Furthermore even though uptake of the core elements of hospital stewardship has increased to 95% across acute care hospitals in the US, there is variation in the way individual programs implement stewardship interventions (e.g., implement antibiotic guidelines) [30–32] and different strategies have different levels of success and can result in differences in guideline concordance at the hospital level.

While our study adds important context to the antibiotic treatment of CAP, it does have limitations. As a retrospective observational study, causality cannot be inferred. We obtained data from a large de-identified database and were limited in the type of data and granularity of data available. We were limited to the use of ICD-10 codes for diagnoses and it is possible some patients were misclassified and as a result inappropriately included or excluded from our sample. Additionally we did not have information on behavioral data such as smoking and alcohol use, which limited our comparison to other studies that accounted for sociodemographic factors. We were unable to examine time to treatment and did not have access to medication post-discharge and thus could not examine duration of therapy as part of our definition of guideline concordant treatment. This may have contributed to differences in findings between our study and other published literature as racial and ethnic differences may exist across some metrics of guideline concordance (e.g., time to treatment) but not others. We were also unable to examine timing of infections acquired during hospitalization or time of death. Although we assessed ICU admissions, clinical factors that may have differed by race/ethnicity such as CAP severity including validated measures such as the pneumonia severity index (PSI) or results from culture and imaging were not available. These clinical factors could affect antibiotic choice, however we did not have access to data that would allow us to make that determination. Additionally severity of disease including the PSI has been shown to be associated with clinical outcomes such as mortality [33] but we were unable to account for this in our analyses. Data in the Vizient Clinical Data Base are voluntarily uploaded and only those hospitals that uploaded their data during the study period were included. It is possible sites that did not upload their data differ from those that did and may serve different types of patients and may be located in different geographic areas. Additionally, our sample only included admissions with a diagnosis of CAP and so we could not account for demographics for the full inpatient cohort and thus could not determine if hospitals served predominantly Black, White, Hispanic or Asian patients. There are several other factors, such as local resistance patterns and hospital-specific stewardship guidelines that can influence antibiotic treatment choices that we were unable to account for in this study because we did not have access to that data. Finally this analysis included admissions that occurred during the COVID-19 pandemic which had widespread impacts on healthcare facilities. The healthcare workforce experienced significant attrition in part due to increased work burden and the mental health toll of the pandemic [34,35]. Healthcare workers also had to learn new ICD-10 codes for COVID-19 and COVID-19 pneumonia that may have led to misclassification of some patients in our sample during the study period. Additionally the uncertainty around diagnosis and treatment of COVID-19 often resulted in unnecessary use of antibiotics [36]. Although we did not include COVID-19 pneumonia diagnoses in our analysis to reduce complications with treatment decisions that providers faced during the pandemic, our results show increasing antibiotic guideline discordance from 2018–2021 which was likely due to many pandemic related disruptions and overall trends in antibiotic use during the pandemic [37]. To account for the impact pandemic related disruptions we adjusted for year in our final model.

Even in the presence of these limitations, our study has several strengths. Our study represents a large sample of patients from a geographically diverse swath of hospitals across the US with robust data on patient race and ethnicity. We were able to build models that included both patient-level and hospital-level factors that may affect antibiotic treatment and clinical outcomes in patients with CAP, thus providing a more comprehensive examination of the factors at play. For example some existing studies do not account for underlying patient comorbidities and miss the role that medical complexity plays in provider antibiotic choice [20,19].The ability to capture both patient and hospital level factors in statistical analyses is especially important in datasets with diverse types of hospitals with different levels of resources which may impact documentation and incompletely reflect the patient population served [38,39]. Additionally, most prior studies were conducted in the outpatient setting and few were done in the inpatient setting where differences in prescribing may have more adverse outcomes. For example, a recent systematic scoping review on health equity an antibiotic prescribing in the United States comprising 61 studies highlighted among other factors, differences by patient race and ethnicity, socioeconomic factors and geography and concluded that differences were likely representative of structural inequities [40]. However that review included only one acute care setting which further highlights the need for research in the inpatient setting [40].

## Conclusion

Our work adds important nuance to the ongoing discussion about the role of both individual and system level factors that can result in differences in antibiotic treatment. Our results, taken together with the existing published literature, point to the fact that patient medical complexity as well as the underlying resources of the hospital and hospital culture are driving factors in antibiotic treatment. Given the existing literature on differences in antibiotic treatment in hospitals that serve a predominantly Black population, our findings further indicate that additional research is needed to understand how the interplay of patient and hospital level factors translate to treatment differences at the individual level.

## Supporting information

S1 Table Description of Hospital Level Characteristics by Region. (DOCX)

S2 Table Full regression results from the final model for the association between race/ethnicity and receipt of guideline concordant care. (DOCX)
