# Supplementary material for: Are there racial/ethnic differences in antibiotic treatment of community acquired pneumonia in the inpatient setting?
Source: PLoS One. 2026 Mar 25;21(3):e0345788. doi: 10.1371/journal.pone.0345788 (PMC13016333; doi:10.1371/journal.pone.0345788)
Supplement: S1 Table — (DOCX) [file pone.0345788.s001.docx]

|  | **Midwest n=169 (36.98%)** | **Northeast n=70 (15.32%)** | **South n=131 (28.67%)** | **West n=87 (19.04)** |
| --- | --- | --- | --- | --- |
| **AMMC Hospital** | 33 (19.53) | 32 (45.71) | 41 (31.3) | 22 (25.29) |
| **Non-AAMC Hospital** | 136 (80.47) | 38 (54.29) | 90 (68.7) | 65 (74.79) |
| **Number of hospital beds/ n (%)** |  |  |  |  |
| <75 | 47 (27.81) | 5 (7.14) | 23 (17.56) | 27 (31.03) |
| 75-199 | 48 (28.4) | 17 (24.29) | 34 (25.95) | 17 (19.54) |
| 200-499 | 45 (26.63) | 21 (30) | 32 (24.43) | 28 (32.18) |
| >=500 | 29 (17.16) | 27 (38.57) | 42 (32.06) | 15 (17.24) |
| **Case Mix Index/ n (%)** |  |  |  |  |
| <1.6 | 55 (32.54) | 16 (22.86) | 24 (18.32) | 17 (19.54) |
| 1.6-1.8 | 45 (26.63) | 14 (20) | 32 (24.43) | 17 (19.54) |
| 1.81-2 | 25 (14.79) | 14 (20) | 23 (17.56) | 19 (21.84) |
| >2 | 44 (26.04) | 26 (37.14) | 52 (39.69) | 34 (39.08) |
