# Supplementary material for: Are there racial/ethnic differences in antibiotic treatment of community acquired pneumonia in the inpatient setting?
Source: PLoS One. 2026 Mar 25;21(3):e0345788. doi: 10.1371/journal.pone.0345788 (PMC13016333; doi:10.1371/journal.pone.0345788)
Supplement: S2 Table — (DOCX) [file pone.0345788.s002.docx]

| **Variable** | |  |  |  |
| --- | --- | --- | --- | --- |
| **Race/Ethnicity** | Adjusted Odds Ratio (95% Confidence Interval) | Estimate | Standard Error | p value |
| Non-Hispanic White | **Reference** |  |  |  |
| Non-Hispanic Black | 1.22 (1.21-1.23) | 0.1965 | 0.006 | <.0001 |
| Asian | 1.00 (0.98-1.03) | 0.0017 | 0.012 | 0.89 |
| Hispanic | 1.15 (1.14-1.17) | 0.1431 | 0.009 | <.0001 |
| Other | 1.05 (1.03-1.07) | 0.0468 | 0.011 | <.0001 |
| **Cerebrovascular disease** | 0.71 (0.70-0.72) | -0.3459 | 0.008 | <.0001 |
| **Renal failure** | 0.76 (0.76-0.77) | -0.2677 | 0.004 | <.0001 |
| **Number of hospital beds** |  |  |  |  |
| <75 | **Reference** |  |  |  |
| 75-199 | 0.94 (0.92-0.96) | -0.05817 | 0.011 | <.0001 |
| 200-499 | 0.81 (0.80-0.83) | -0.2035 | 0.011 | <.0001 |
| >=500 | 0.68 (0.66-0.69) | -0.3864 | 0.012 | <.0001 |
| **Case Mix index** |  |  |  |  |
| <1.6 | **Reference** |  |  |  |
| 1.6-1.8 | 0.84 (0.83-0.85) | -0.173 | 0.01 | <.0001 |
| >1.8-2 | 0.93 (0.92-0.95) | -0.0679 | 0.01 | <.0001 |
| >2 | 0.71 (0.69-0.72) | -0.3436 | 0.01 | <.0001 |
| **Hospital Region** |  |  |  | <.0001 |
| West | **Reference** |  |  |  |
| South | 0.8 (0.79-0.81) | -0.2229 | 0.006 | <.0001 |
| Midwest | 0.83 (0.81-0.83) | -0.1948 | 0.006 | <.0001 |
| Northeast | 0.71 (0.70-0.72) | -0.3432 | 0.007 | <.0001 |
| **Year** |  |  |  |  |
| 2018 | **Reference** |  |  |  |
| 2019 | 0.87 (0.86-0.88) | -0.1414 | 0.005 | <.0001 |
| 2020 | 0.80 (0.79-0.81) | -0.2235 | 0.005 | <.0001 |
| 2021 | 0.73 (0.73-0.74) | -0.3096 | 0.005 | <.0001 |
